# Potentiation of *in vitro* and *in vivo* antitumor efficacy of doxorubicin by cyclin-dependent kinase inhibitor P276-00 in human non-small cell lung cancer cells

**DOI:** 10.1186/1471-2407-13-29

**Published:** 2013-01-23

**Authors:** Maggie J Rathos, Harshal Khanwalkar, Kavita Joshi, Sonal M Manohar, Kalpana S Joshi

**Affiliations:** 1 Oncology Franchise, Piramal Healthcare Limited, 1-Nirlon Complex, Goregaon, Mumbai, 400 063, India; 2 Target Identification Group, Piramal Healthcare Limited, 1-Nirlon Complex, Goregaon, Mumbai, 400 063, India

**Keywords:** Non-small cell lung cancer, P276-00, Doxorubicin, Cyclin-dependent kinase inhibitor, Chemotherapy

## Abstract

**Background:**

In the present study, we show that the combination of doxorubicin with the cyclin-dependent kinase inhibitor P276-00 was synergistic at suboptimal doses in the non-small cell lung carcinoma (NSCLC) cell lines and induces extensive apoptosis than either drug alone in H-460 human NSCLC cells.

**Methods:**

Synergistic effects of P276-00 and doxorubicin on growth inhibition was studied using the Propidium Iodide (PI) assay. The doses showing the best synergistic effect was determined and these doses were used for further mechanistic studies such as western blotting, cell cycle analysis and RT-PCR. The *in vivo* efficacy of the combination was evaluated using the H-460 xenograft model.

**Results:**

The combination of 100 nM doxorubicin followed by 1200 nM P276-00 showed synergistic effect in the p53-positive and p53-mutated cell lines H-460 and H23 respectively as compared to the p53-null cell line H1299. Abrogation of doxorubicin-induced G2/M arrest and induction of apoptosis was observed in the combination treatment. This was associated with induction of tumor suppressor protein p53 and reduction of anti-apoptotic protein Bcl-2. Furthermore, doxorubicin alone greatly induced COX-2, a NF-κB target and Cdk-1, a target of P276-00, which was downregulated by P276-00 in the combination. Doxorubicin when combined with P276-00 in a sequence-specific manner significantly inhibited tumor growth, compared with either doxorubicin or P276-00 alone in H-460 xenograft model.

**Conclusion:**

These findings suggest that this combination may increase the therapeutic index over doxorubicin alone and reduce systemic toxicity of doxorubicin most likely via an inhibition of doxorubicin-induced chemoresistance involving NF-κB signaling and inhibition of Cdk-1 which is involved in cell cycle progression.

## Background

Lung cancer affects more than 1.2 million patients a year. The prognosis of lung cancer is very poor and long-term survival is obtained in only 5-10% of the patients. Non-small cell lung cancer (NSCLC) constitutes approximately 85% of all lung cancers and is the leading cause of tumor-related death worldwide highlighting the need for more effective treatment strategies [1,2]. NSCLC are inherently resistant and are generally not responsive to initial chemotherapy [3].

Treatment of advanced NSCLC with anthracyclines, exemplified by doxorubicin, provides an overall response rate of only 30–50%. Unfortunately, its acute and cumulative dose-related toxicity poses a major problem in therapeutic outcomes. In addition to toxicity, the development of inducible drug resistance is a paramount problem in which patient fails to respond to cancer drugs.

There are various novel therapeutic strategies currently under consideration for lung cancer, as the clinical use of cytotoxic drugs is limited due to intrinsic or acquired resistance and toxicity. A better understanding of the molecular mechanisms of cytotoxic drug action has shed light on the treatment of lung cancer, and novel agents that target specific intracellular pathways related to the distinctive properties of cancer cells continue to be developed [4]. Most DNA-damaging agents, including doxorubicin, trigger cell death via activation of p53 [5]. The apoptotic role of p53 likely resides in its ability to disrupt the balance between antiapoptotic proteins (such as Bcl-XL, Bcl-2, and Mcl-1) and pro-apoptotic proteins (such as Bax and Bak) [6]. Mee et al. (2008) have shown that p53 is upstream of NF-κB-mediated pathways of doxorubicin resistance and p53 is required for NF-κB mediated resistance to doxorubicin in NSCLC.

Since dysfunctions in the regulation of the cell cycle were found in almost all human cancers including NSCLC, agents targeting proteins involved in the regulation of cell cycle progression were developed [7]. Disruption of the Rb pathway is a frequent event in NSCLC and plays an important role in tumorigenesis of NSCLCs [8]. It has been hypothesized that the aberrant expression of cyclin D1 has strong oncogenic activity independently of pRb and p16, and may override the suppressive effects of pRb and p16. Other groups have also shown that the deregulation of cyclin D1 expression is an important characteristic of this disease [9]. These data further strengthen the argument that cyclin D1 might be an effective lung cancer therapeutic target.

P276-00 is a novel small molecule inhibitor of cyclin-dependent kinases (Cdks) and has demonstrated synergism with different chemotherapeutic agents and is in Phase I/II clinical trials in combination with such agents/radiation for various cancers [10]. In this study, we investigated the effect of P276-00, doxorubicin and their combination in three NSCLC cell lines differing in their p53 status and their *in vivo* antitumor efficacy against a human NSCLC (H-460) xenograft.

## Methods

### Cell culture and reagents

Human NSCLC cell lines H-460 (p53-positive), H1299 (p53-null) and H23 (p53-mutant) were obtained from ATCC (Rockville, MD, USA) and cultured in RPMI-1640 medium containing 10% fetal bovine serum (FBS) (Hyclone, UT, USA), 2 mmol/L L-glutamine (Gibco, Grand Island, NY, USA), 100 U/mL penicillin and 100 mg/mL streptomycin (Gibco). Cells were maintained at 37°C in a humidified atmosphere containing 5% CO_2_. P276-00 was synthesized at Piramal Healthcare Limited, Mumbai, India and doxorubicin was purchased from Sigma. Both drugs were dissolved in dimethyl sulfoxide (DMSO) at a concentration of 10 mmol/L (10 mM) and stored at −20°C until use; diluted in culture medium RPMI-1640 immediately before use and used within 4 h. All reagents were purchased from Sigma (St. Louis, MO, USA) unless stated otherwise.

### *In vitro* cytotoxicity assay

Cells were plated in 96-well plates and allowed to attach overnight. Each concentration of doxorubicin and P276-00 was represented by 3 wells per experiment and each experiment was repeated three times. Treated cells were maintained at 37°C in 5% CO_2_ for times indicated in the legends to the figures. A modified propidium iodide (PI) assay was used to assess the effect of the compounds on the growth of the human tumor cell lines [11]. IC_50_ values were determined by plotting compound concentration versus cell viability. The combination index (CI) was calculated by the Chou-Talalay equation, which takes into account both the potency and the shape of the dose-effect curve taking advantage of the Compusyn software (ComboSyn, Inc. NY, USA). The combination index is used for the quantification of synergism or antagonism for two drugs where CI< 1, =1, and >1 indicate synergism, additive effect, and antagonism, respectively.

### Analysis of cell cycle distribution by flow cytometry

H-460 cells were seeded in T-25 tissue culture flasks at a density of 1.0 × 10^6^/mL and incubated overnight at 37°C. Next day the cells were treated with compounds. At the end of treatment period the cells were harvested and processed for flow cytometry as described previously [11].

### Annexin V staining

Annexin V staining was performed as described by the manufacturer (BD Biosciences). Briefly, 1 × 10^5^ cells were washed twice in PBS and resuspended in binding buffer (10 mM HEPES, NaOH (pH 7.4), 140 mM NaCl, 2.5 mM CaCl_2_) at a concentration of 1 × 10^6^ cells/ml. 5 μl of FITC-Annexin V (BD Biosciences) and 10 μl of PI (500 μg/ml in 38 mM sodium citrate) were added, and the cells incubated for 15 min in the dark at room temperature. A total of 400 μl of binding buffer was then added and the cells analyzed by flow cytometry.

### Preparation and analysis of cell lysates by immunoblotting

Cells were seeded, treated with or without P276-00 or doxorubicin or their combination and were harvested at desired time points and western blotting was carried out as previously described [12]. Antibodies used in this study were: Bcl-2, Bax, p53, Cdk-1, cyclin D1 (Santacruz Biotechnology, CA, USA), Cox-2 (Cell signaling technology, USA), anti-rabbit–HRP and anti-mouse-HRP secondary antibodies (Santacruz Biotechnology, CA, USA).

### Clonogenic assay

H-460 cells were seeded at a density of 750–1000 cells per 35 mm tissue culture grade plate and incubated overnight at 37°C for the cells to attach. The cells were treated with the cytotoxic drug doxorubicin for 24 h followed by removal of medium and addition of fresh medium containing P276-00 for 96 h. At the end of the treatment, the medium was replaced by fresh complete medium and incubated for 7–14 days for colony formation. When visible colonies appeared on the plate the medium was removed and colonies were fixed with methanol: acetic acid mixture in the ratio of 2:1 for 5 min. The plates were washed with water and the fixation procedure was repeated. The plates were dried and the colonies were stained with 0.1% crystal violet stain for 3–5 min. The plates were rinsed carefully with water and dried and the colonies counted.

### Tumor xenograft model

Approximately 5 × 10^6^ H-460 cells were subcutaneously injected into severe combined immunodeficient (SCID) mice in 0.2 ml volume on the right flank and observed daily for tumor appearance. When the tumors attained a diameter of ~50 mm^3^, they were randomized into four groups: Group I, control vehicle; Group II, doxorubicin 2 mpk ip. once a week for 2 weeks; Group III, P276-00 20 mpk ip. every day for 5 days a week for 2 weeks; Group IV, combination of doxorubicin and P276-00, doxorubicin was followed by P276-00 after an interval of 6 h, followed everyday with P276-00 for a total of five days which comprised of one cycle. The treatment comprised of total of two cycles. Body weight was recorded everyday. Tumor measurements i.e. the length and width of the tumors were measured using the vernier caliper. Tumor weight (mg) was estimated according to the formula for a prolate ellipsoid: {Length (mm) x [width (mm)^2^] x 0.5} assuming specific gravity to be one and π to be three. Tumor growth in compound treated animals is calculated as T/C (Treated/Control) x 100% and growth inhibition percent (GI %) was [100-T/C%]. Animals were maintained and experiments were carried out as per the institutional animal ethical committee in compliance with the guidelines of the Committee for the Purpose of Control and Supervision on Experiments on Animals (CPCSEA), India.

### Statistical analysis

Statistical comparison was made using GraphPad Prism software (version 5.0) in which one-way analysis of variance and Tukey’s multiple comparison post tests were used to determine significant differences between several treatment groups. Student’s paired *t*-test was used when only two groups were compared. Data are presented as mean ± S.E.M. of at least three independent experiments with triplicate. Statistical significance was evaluated by calculating *P*-values. Differences where *P* < 0.05 were considered statistically significant (^*^P < 0.05; ^**^P < 0.01; ^***^P < 0.001).

## Results

### Effect of P276-00 and doxorubicin on cell proliferation

**Figure 1:**
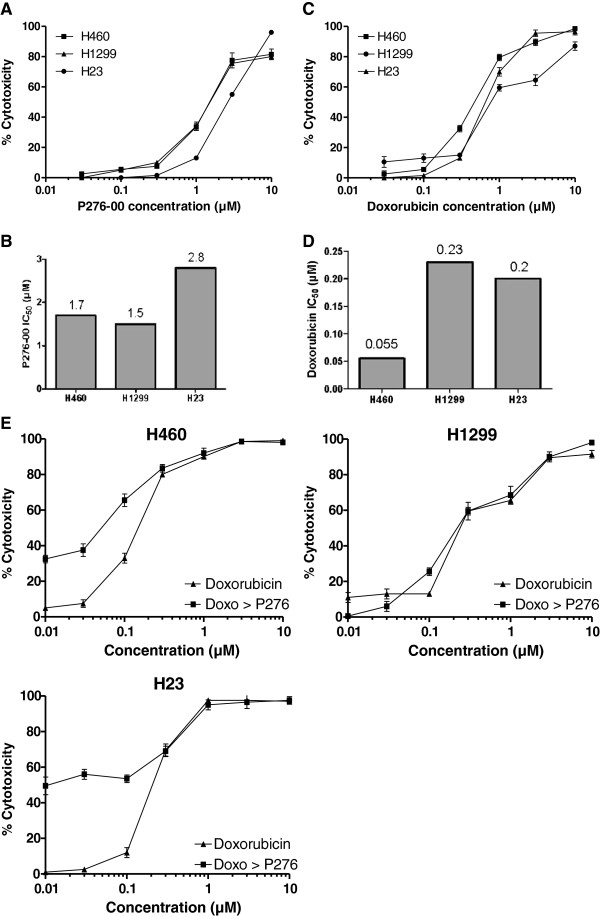
**(A & B) The effect of P276-00 and doxorubicin treatment on growth of three NSCLC cell lines.** Cells were seeded in 96-well plates and incubated overnight. P276-00 was added at the indicated concentrations and cells were further incubated for 48 h. Cell proliferation was determined using PI assay. **(C** &**D)** The 50% inhibitory concentrations of P276-00 and doxorubicin in three different NSCLC cell lines. **(E)** Effect of P276-00 and doxorubicin used singly or in combination on survival of H-460, H1299, H23 cell lines. The cells were treated as described under Materials and method section. There was significantly higher growth inhibition of the cells treated with doxorubicin (24 h) followed by P276-00 at IC_50_ concentration for 72 h compared to the cells treated with either agent alone.

Three NSCLC cell lines H-460, H1299 and H23 were treated with increasing concentrations (0.01-10 μM) of P276-00 for 72 h and cell proliferation was assessed. Treatment with P276-00 caused a dose-dependent decrease in the proliferation of all the three cell lines (Figure 1A). Thus, P276-00 was an effective inhibitor of NSCLC cancer cell growth as a single agent, and H-460 and H1299 were more sensitive than H23 as seen from the IC_50_ values (Figure 1B). Doxorubicin was also effective as a single agent and H-460 was highly sensitive to doxorubicin while H1299 and H23 were moderately sensitive (Figure 1C and 1D).

### P276-00 potentiates growth inhibition induced by doxorubicin in various human NSCLC cell lines

The anthracycline antibiotic doxorubicin is commonly used in lung cancer treatment regimens. However, in contrast to its high activity in SCLC, doxorubicin is not curative in NSCLC which represents 4/5 of all lung cancers [13]. To determine if P276-00 enhances the sensitivity of NSCLC cells to the growth inhibitory effect of doxorubicin, combination studies were done. Cells were either treated with P276-00 (IC_50_ concentration) or doxorubicin or in combination with serial concentrations of doxorubicin (0.01-10 μM) followed by P276-00 (IC_50_ concentration) for 72 h and cell viability was evaluated. Combination treatment yielded significantly greater growth inhibition in a dose-dependent manner than either agent alone in all three cell lines (Figure 1E). The combination index method developed by Chou [14] was used to confirm and quantify the synergism observed with doxorubicin and P276-00. The CI values of the combination of IC_50_ of P276-00 with various concentration of doxorubicin were calculated using CompuSyn software. The p53 positive H-460 and p53 mutant H-23 cell lines showed synergism with CI range of 0.63-0.94 and 0.86-0.9 respectively. The combination of doxorubicin followed by P276-00 was not synergistic in the p53 null H1299 cell line. For all further studies, H-460 cell line was selected, which possesses wild type *p53* gene, mutant *KRAS* and wild type *EGFR*[15].

### Synergistic cytotoxicity of doxorubicin and P276-00 combination is due to increased apoptosis

**Figure 2:**
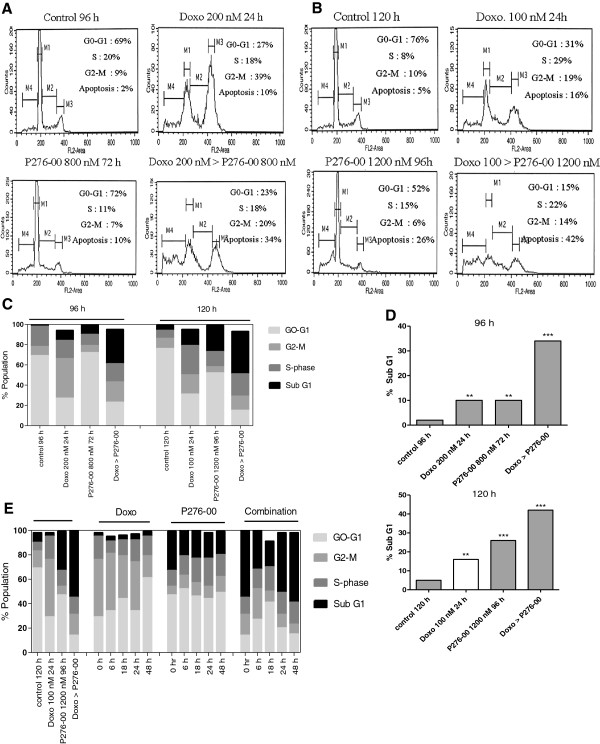
**The effect of P276-00, doxorubicin and the combination of doxorubicin followed by P276-00 on cell proliferation, cell cycle and on recovery of cells after the treatment.(A)** Cells were treated with 200 nM doxorubicin for 24 h or 800 nM of P276-00 for 72 h or combination of both. **(B)** Cells were treated with 100 nM doxorubicin for 24 h or 1200 nM P276-00 for 96 h or combination of both. **(C)** Percent of cell population in different phases of cell cycle. **(D)** Percent of cell population in sub-G1 phase after treatment with either drug alone or combination for 96 h and 120 h and **(E)** Recovery of cells after the drug treatment was studied at different timepoints i.e. 0 h, 6 h, 18 h, 24 h and 48 h. Cell cycle analysis using Flow cytometry shows the percentage apoptosis in drug alone and combination treatment in comparison to untreated control.

We observed induction of apoptosis in H-460 cells treated with either doxorubicin or P276-00 or both (Figure 2A, 2B and 2C). Relative to single agents, the combination treatment induced more apoptosis as shown in Figure 2C and 2D (*P* < 0.001). In fact, doxorubicin induced G2/M arrest, which was overcome by subsequent P276-00 treatment. These results are consistent with cell growth inhibition studies. The combination of 100 nM doxorubicin (24 h) followed by 1200 nM (IC_50_) of P276-00 for 72 h was found to be the most synergistic and hence used for further mechanistic studies.

### NSCLC cells continue to undergo apoptosis in combination treatment even after the drugs are removed

#### Recovery experiment

In this experiment, cells were first treated with either doxorubicin (24 h) or P276-00 (96 h) or combination of both. After the treatment, drugs were removed and cells were further incubated in fresh medium without drug treatment. As indicated in Figure 2E at the end of 120 h P276-00 and doxorubicin showed 32 and 3% of apoptosis respectively, whereas combination showed increased apoptosis of 55%. At this point one set of samples viz. control, P276-00, doxorubicin and combination was analyzed for early induction of apoptosis using Annexin V binding assay and the second set of same samples was incubated with fresh medium and analyzed by flow cytometry at 6, 18, 24 and 48 h time points. It was observed that doxorubicin did not induce further apoptosis, while the combination showed the greatest increase in apoptosis of 57% by the end of 48 h indicating that cells in the combination treatment continued to undergo apoptosis.

**Figure 3:**
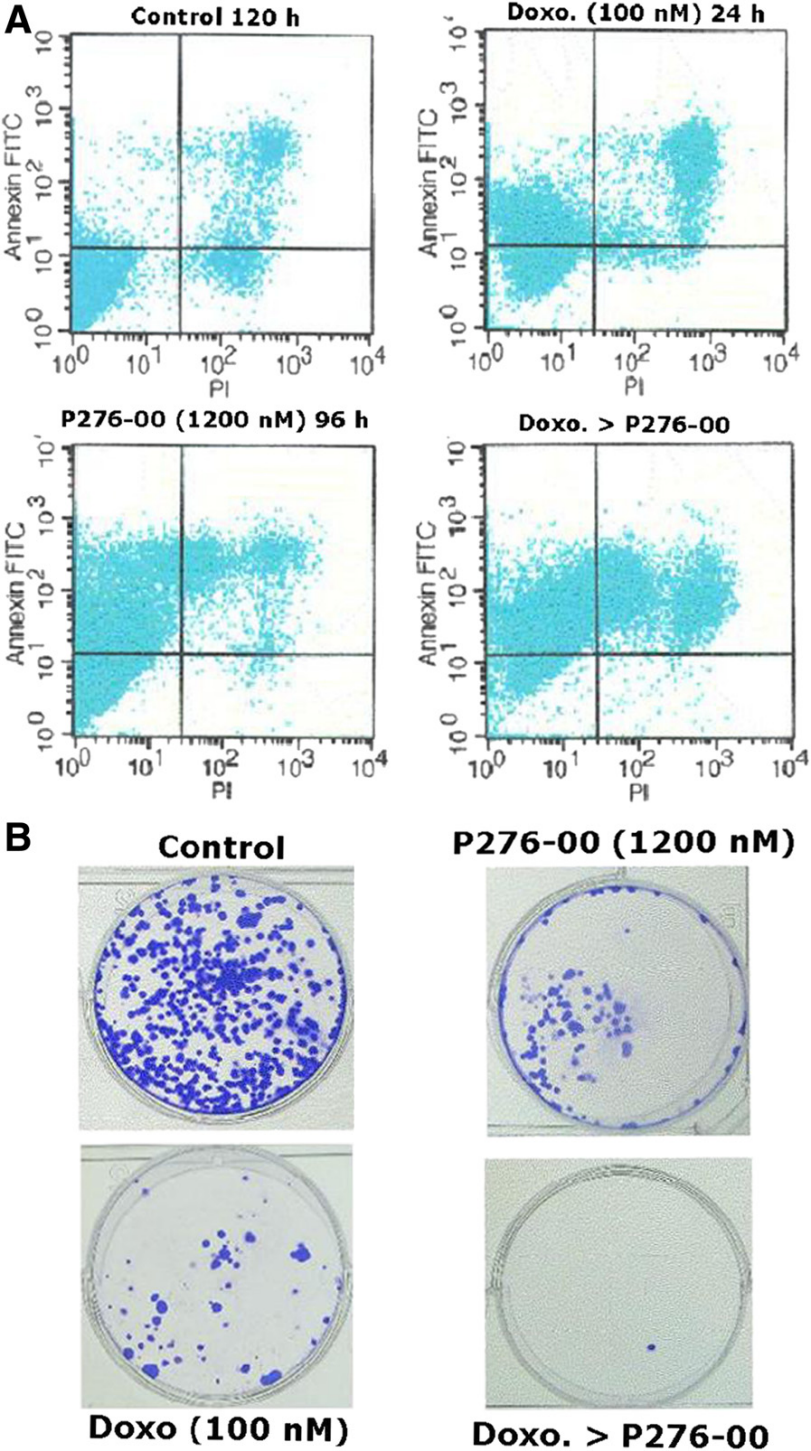
(**A**) **Annexin-V staining for detection of apoptosis and (B) Clonogenic assay in H-460 cells after treatment with doxorubicin alone (100 nM for 24 h) or P276-00 (1200 nM for 96 h) alone or combination of doxorubicin (24 h) > P276-00 (96 h).**

One of the early changes during apoptosis is loss of plasma membrane asymmetry that exposes phosphatidylserine on the cell surface. This process precedes loss of plasma membrane integrity and can be detected by Annexin V binding. Annexin V binding analysis after the combination treatment confirmed that this treatment effectively induced apoptosis leading to phosphoserine externalization (Figure 3A). 34.3% cells stained by both Annexin and PI had already undergone apoptosis in the combination as compared to either drug alone (~8%). Interestingly 50.2% of the population was Annexin-V positive indicating that the cells had already entered apoptosis when fresh medium was added and hence these cells continued to undergo apoptosis and did not recover. Therefore, in combination after medium change, total 84.5% (50.2 + 34.2%) cells are either just entering apoptosis or in the early stages of apoptosis verses ~40% for either drug alone (Table 1). Cytotoxicity assay and flow cytometry were complemented with the conventional clonogenic assay in H-460. It demonstrates a significant synergistic effect between both the drugs (Figure 3B), as seen from the number of colonies in the combination as compared to drug alone.

**Table 1:** Percentage of H-460 cells in late or early apoptosis at the end of treatment period either with doxorubicin or P276-00 alone or in the combination of doxorubicin followed by P276-00

**Treatment group**	**Percentage of cells**			
	**Live cells**	**Annexin +ve**	**Annexin + PI**	**PI +ve**
Control	90.5	3	4	2.3
Doxorubicin ( 100 nM)	60	30.4	8.6	1
P276-00 (1200 nM)	53	38.5	8.2	0.3
Doxorubicin> P276-00	14.1	50.2	34.3	2

### Effect of combination of P276-00 and doxorubicin on cell cycle related and antiapoptotic proteins

**Figure 4:**
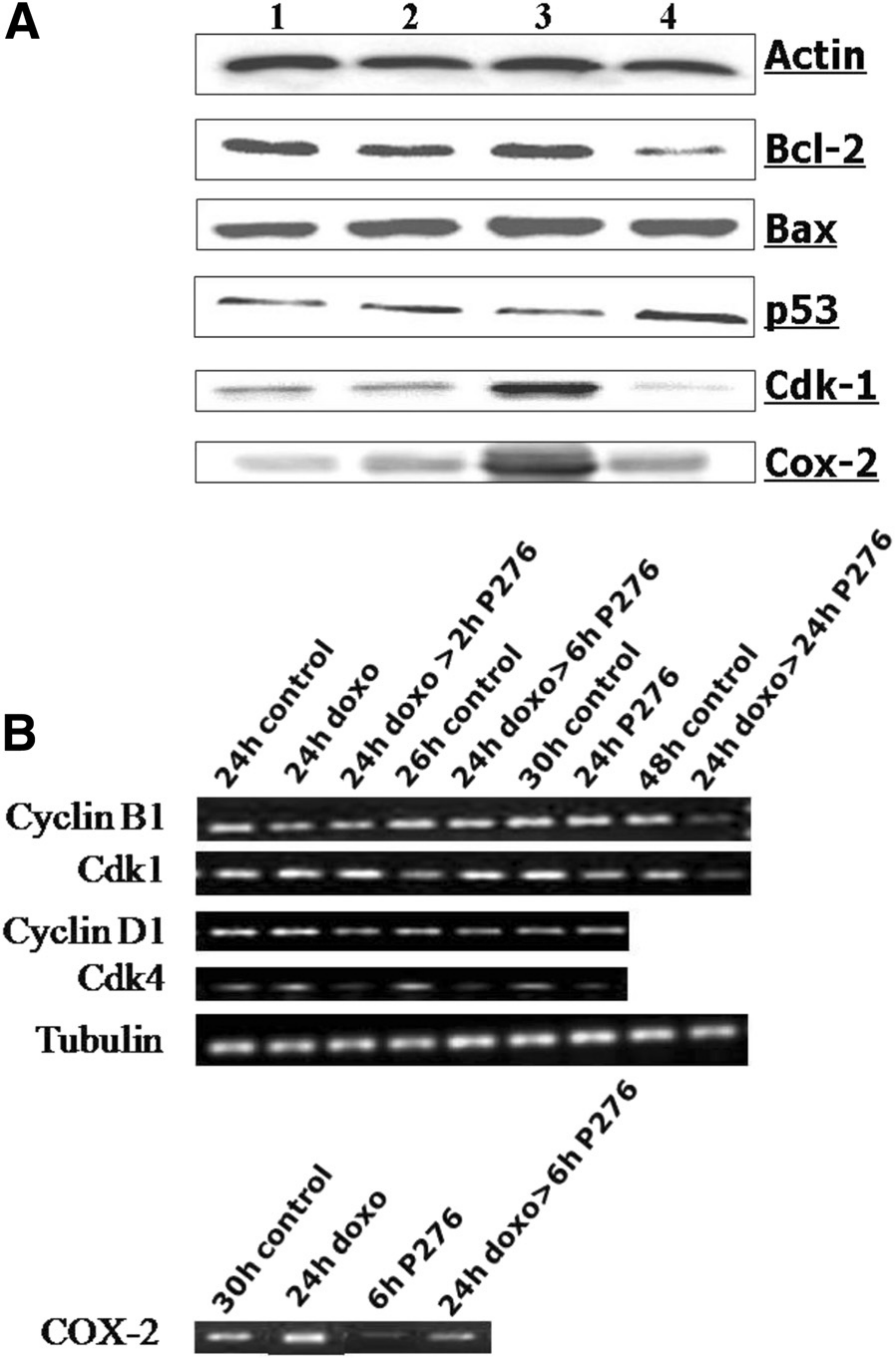
**RT-PCR and Western blot analysis in H-460 cells. (A)** Effect of combination of P276-00 and doxorubicin or either of the drugs alone on the protein levels 1. Control, 2. P276-00, 3. doxorubicin, 4. doxorubicin> P276-00. **(B)** mRNA analysis of cyclin B1, Cdk-1, cyclin D1, Cdk-4 and Cox-2. Samples were obtained from H-460 cells treated with 100 nM of doxorubicin and/or 1200 nM P276-00 at various time points as indicated in the figure.

Next, we analyzed whether combination treatment could inhibit the expression of cell cycle related proteins and antiapoptotic proteins that are modulated by doxorubicin and could be involved in chemoresistance (Figure 4A). Cdk-1 levels that were upregulated by doxorubicin treatment were inhibited when followed by P276-00 exposure. Additionally p53 was significantly increased and Bcl-2 was decreased after combination treatment but not with either agent alone. There was no significant change in Bax levels on treatment with either drug alone or with combination.

Earlier studies have indicated that NF-κB activation plays an important role in inducible chemoresistance to anthracycline drugs in many cancer cells [16]. COX-2 has been shown as a target gene for NF-κB and implicated in lung cancer growth. Protein and gene expression studies indicated that P276-00 downregulated protein levels of COX-2 which were upregulated after doxorubicin treatment (Figure 4A). Gene expression levels of both Cdk-1 and its cyclin partner cyclin B1 are significantly downregulated at 24 h post P276-00 treatment. However, moderate decrease in cyclin D1 and Cdk4 levels was observed at 2 and 6 h post P276-00 treatment (Figure 4A). The densitometry plots of the RT-PCR bands are shown in Additional file 1: Figure S1.

### P276-00 potentiates anti-tumor effect of doxorubicin in xenograft model of NSCLC

**Figure 5:**
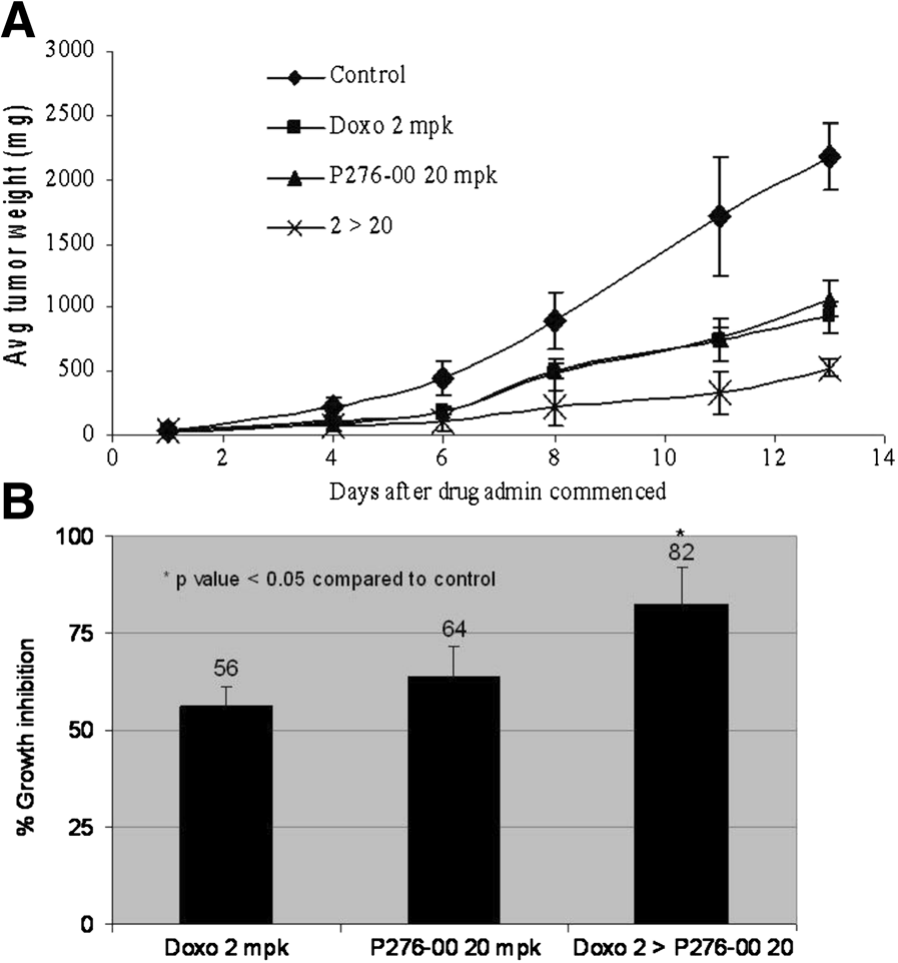
**The combination treatment of doxorubicin with P276-00 showed significant tumor growth inhibition in H-460 xenograft model. (A)** Differences in the tumour weight (mg). **(B)** Percent growth inhibition after treatment. Statistically significant difference (*P* <0.05) of the combination treatment of doxorubicin and P276-00 compared with the control was seen.

The effects of the combination on human NSCLC H-460 tumor xenograft was studied to determine if the synergy observed *in vitro* between P276-00 and doxorubicin also occurred *in vivo*. Treatment with either P276-00 (20 mpk once daily) or doxorubicin (2 mpk once a week) was initiated when tumors reached a size of ~50 mm^3^ in diameter. P276-00 and doxorubicin alone caused significant suppression of tumor growth, while the combination of the two drugs showed highly significant reduction in the mean tumor weight (Figure 5A). Tumor growth inhibition of 82% was seen at the end of the treatment period as compared to P276-00 (64%) and doxorubicin (56%) (*P* ≤ 0.05) (Figure 5B). No body weight loss was observed in both the combination and single drug treated groups indicating that the doses and schedule were well tolerated.

## Discussion

We evaluated the cytotoxic effects of either doxorubicin or P276-00 or the combination of both the compounds in three NSCLC cell lines viz. H-460 (p53-positive), H1299 (p53-null) and H23 (p53-mutant). A sequential drug treatment strategy was chosen based on previous studies demonstrating sequence-specific synergistic effects with administration of combination of chemotherapy (doxorubicin) and Cdk inhibitor P276-00 (data not shown). The combination was synergistic in the p53 positive and p53 mutant cell line but not p53 null cell line indicating that p53 may possibly have a role in the synergistic interaction. Similar results have been observed with combined treatment of doxorubicin and another Cdk inhibitor roscovitine in human sarcoma cell lines [17]. It has been shown earlier that doxorubicin mediated cell cycle arrest can occur either at G0/G1 or G2 check points and is thought to be mediated by the multifunctional transcription factor p53 [18]. Doxorubicin is more effective in p53 wild type cancers. Our results were in line with this finding – the best synergism between the two drugs was observed in H-460 (p53-positive) cell line and hence it was selected for all further studies.

Cell cycle analysis demonstrated that doxorubicin alone markedly increased the percentage of cells in the G2/M phase. Same response was seen earlier by other groups in another doxorubicin sensitive human lung carcinoma cell line DLKP-SQ [19]. It has been proposed that the G1 checkpoint is disabled in majority of cancers as a prerequisite for tumorigenesis. The G2 checkpoint, however, appears to remain functional in the majority of cancer cells. Many conventional cancer therapeutic agents exert their effect by causing DNA damage and thus retaining a functional G2 is believed to confer resistance to many such therapeutic agents. To circumvent this, attempts have been made to develop abrogators of this arrest in the G2 phase. Doxorubicin is also known to cause G2 arrest in several cell lines and the target for the G2 checkpoint pathway is Cdk1 [20]. Combination treatment was found to downregulate Cdk1 expression levels which could account for abrogation of G2/M arrest and induction of apoptosis.

The checkpoint inhibitor p16 is practically always silenced in NSCLC due to methylation of its promoter [21]. Loss of p16 expression leads to Rb phosphorylation by the cyclin D- cyclin-dependent kinase 4, 6 complex releasing E2F with the onset of the S phase of the cell cycle. Gain of the 11q13.1-11q14.1 region has been shown to be present in > 50% of the lung cancer cell lines. Cyclin D1 is located at this loci and the amplification of this gene is an important event in tumorigenesis [22]. P276-00 is a potent Cdk4 and Cdk1 inhibitor [10] and therefore, downregulation of Cdk4, Cdk1, cyclin D1 and cyclin B1 was observed by P276-00 alone and in combination. This could be a potential factor in the increased sensitivity of H-460 to the combination compared to either drug alone.

Many anticancer agents increase Bax protein and/or decrease Bcl-2 protein during the apoptotic process. Similarly, doxorubicin and P276-00-induced apoptosis in H-460 cells was accompanied by an elevation of the Bax to Bcl-2 ratio due to the downregulation of Bcl-2. p53 induces cell cycle arrest or apoptosis in response to DNA damage and regulates Bax and Bcl-2 protein expression [4]. In response to the combination treatment, p53 levels were significantly upregulated, which could have lead to modulation of Bax and Bcl-2 expression.

One of the targets currently being evaluated in the treatment of lung cancer belongs to the cyclooxygenase (COX) class of enzymes. COX-2 overexpression is seen in many malignancies including lung cancer [1]. Recently, it was shown by O’Kane et al. 2010 [23] that COX-2 specific inhibitors enhance the cytotoxic effect of conventional drugs. Doxorubicin causes the activation of NF-κB in cancer cells, which in turn inhibits apoptosis induced by doxorubicin; cells with increased activity of NF-κB are thus resistant to doxorubicin [24]. P276-00 when added after doxorubicin treatment significantly inhibited COX-2 protein levels which were increased in response to doxorubicin treatment. The synergy observed *in vitro* was also seen in *in vivo* antitumor efficacy studies at well-tolerated doses and schedules. Anticancer efficacy was more pronounced in combination (*P* ≤ 0.05) as compared to either drug alone.

## Conclusions

There are various novel therapeutic strategies under consideration, as the clinical use of cytotoxic drugs is limited due to intrinsic or acquired resistance and toxicity. Recent efforts have focused on identifying novel combinations of anticancer agents with non-overlapping mechanisms of action to obtain enhanced anticancer efficacy and reduced toxicity. Our results provide substantial evidence for the hypothesis that Cdk inhibitor P276-00 enhances doxorubicin-induced killing of NSCLC cells *in vitro* as well as *in vivo* without any significant toxicity especially in p53 positive tumors. This study will potentially provide new approaches to combination anticancer therapy for p53 positive NSCLC.

## Competing interests

The author(s) declare that they have no competing interests.

## Authors’ contributions

MJR designed the experiments and edited the manuscript. HK performed cell cycle studies, western blotting and *in vivo* studies. KJ carried out cell culture, *in vitro* combination studies and RT-PCR. SMM analyzed the data and wrote the manuscript. KSJ has conceptualized the project, written the discussion and edited the manuscript. All the authors except KJ, gave the final approval of the version to be submitted. Unfortunately Ms. KJ is no more.

## Pre-publication history

The pre-publication history for this paper can be accessed here:

http://www.biomedcentral.com/1471-2407/13/29/prepub

## Supplementary Material

Additional file 1 **Figure S1.** Densitometric analysis of mRNA levels Click here for file
