# Supplementary figures and images for: Potentiation of in vitro and in vivo antitumor efficacy of doxorubicin by cyclin-dependent kinase inhibitor P276-00 in human non-small cell lung cancer cells
Source: BMC Cancer. 2013 Jan 23;13:29. doi: 10.1186/1471-2407-13-29 (PMC3635914; doi:10.1186/1471-2407-13-29)

Supplementary Figure 1

Densitometric analysis of mRNA levels


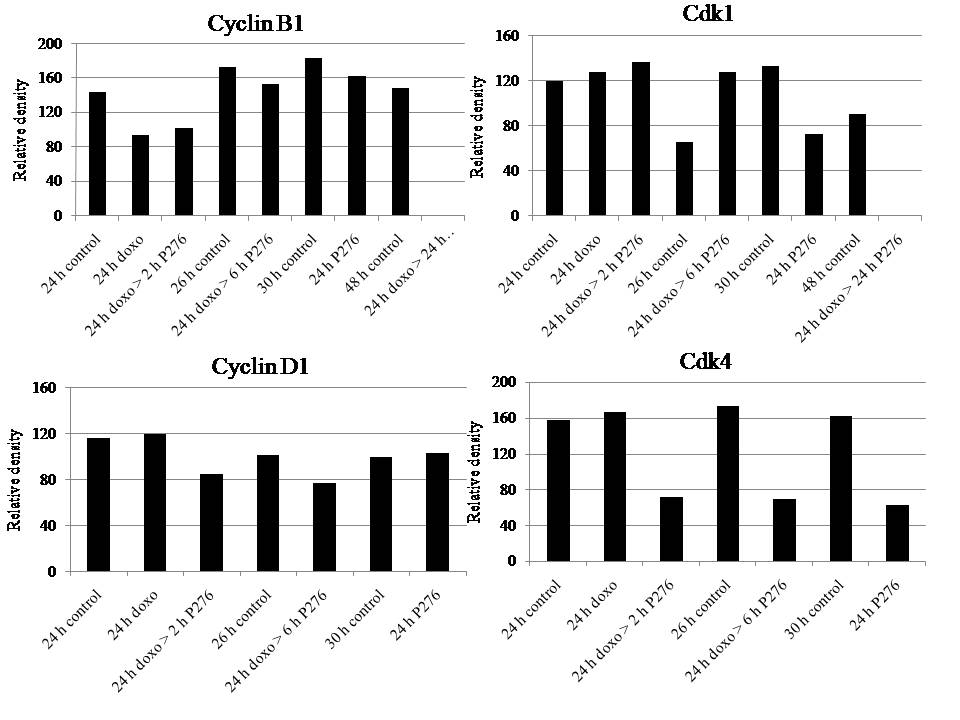


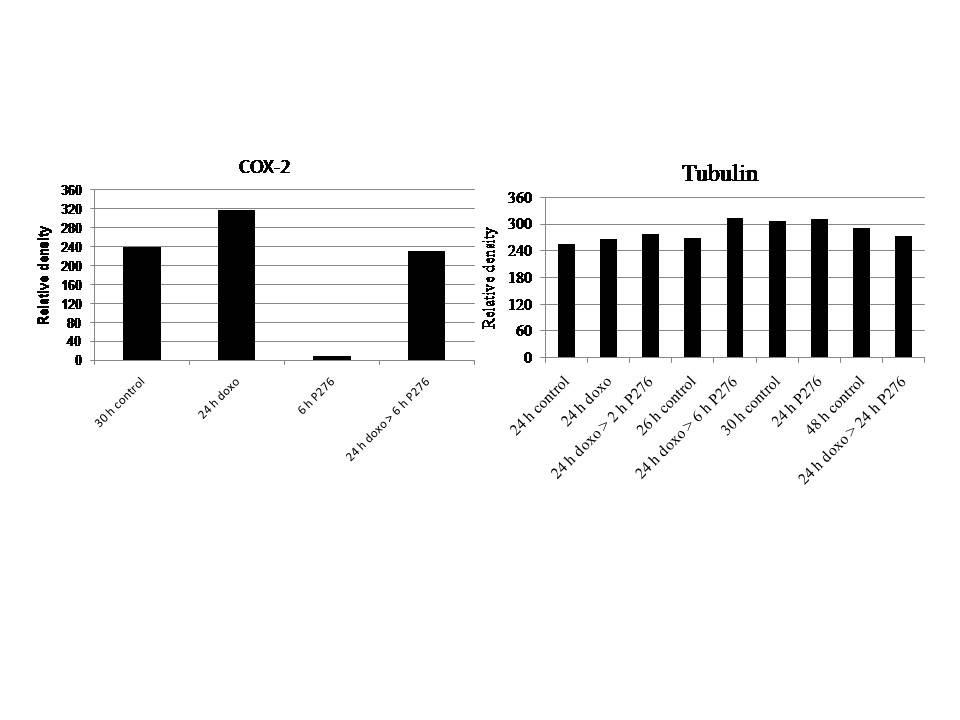

Supplement: Additional file 1 — Figure S1. Densitometric analysis of mRNA levels [file 1471-2407-13-29-S1.doc]
